# Using equilibrium frequencies in models of sequence evolution

**DOI:** 10.1186/1471-2148-5-21

**Published:** 2005-03-02

**Authors:** Bjarne Knudsen, Michael M Miyamoto

**Affiliations:** 1 Department of Zoology, Box 118525, University of Florida, Gainesville, FL 32611-8525, USA

## Abstract

**Background:**

The *f *factor is a new parameter for accommodating the influence of both the starting and ending states in the rate matrices of "generalized weighted frequencies" (+gwF) models for sequence evolution. In this study, we derive an expected value for *f*, starting from a nearly neutral model of weak selection, and then assess the biological interpretation of this factor with evolutionary simulations.

**Results:**

An expected value of *f *= 0.5 (i.e., equal dependency on the starting and ending states) is derived for sequences that are evolving under the nearly neutral model of this study. However, this expectation is sensitive to violations of its underlying assumptions as illustrated with the evolutionary simulations.

**Conclusion:**

This study illustrates how selection, drift, and mutation at the population level can be linked to the rate matrices of models for sequence evolution to derive an expected value of *f*. However, as *f *is affected by a number of factors that limit its biological interpretation, this factor should normally be estimated as a free parameter rather than fixed a priori in a +gwF analysis.

## Background

Felsenstein [1] was the first to introduce an evolutionary model for DNA sequences, which allows for unequal nucleotide frequencies (see also [2]). His F81 model allows for substitutions at a rate proportional to the frequencies of the ending nucleotides. It is considered the simplest rate matrix for accommodating variable nucleotide frequencies and is therefore the starting point for the consideration of more complex models with frequency variation (e.g., the HKY model of Hasegawa *et al*. [3]). Goldman and Whelan [4] described new variants of these F81-based models (their +gwF (generalized weighted frequencies) models; e.g., JC+gwF for Jukes and Cantor [5], and K2P+gwF for Kimura [6]). At the heart of their +gwF variants was a new free parameter (*f*) to accommodate the frequencies of the starting, as well as ending, nucleotides in the evolutionary process:



where *q*_*ij *_refers to the substitution rate from nucleotide *i *to *j*, *π*_*i *_and *π*_*j *_correspond to their equilibrium base frequencies, and *s*_*ij *_is the exchangeability between the two. In the +gwF variants, the substitution rate becomes more dependent on the ending nucleotide as *f *decreases from 1 to 0, with *f *= 0 for the classic F81-type models.

This study starts with a population genetics model to derive equations that link weak selection, genetic drift, and mutation to the *f *parameter and evolutionary rate matrices of the +gwF variants. These theoretical derivations lead to an expected value of *f *= 0.5. However, as illustrated with simulations, the *f *parameter is complex and thus its biological interpretation must be considered with caution.

## Results

### Derivation of the rate matrix for the weak selection model

The nearly neutral model of molecular evolution states that most DNA mutations of longer-term evolutionary consequence are under weak selection and are therefore prone to drift [7,8]. For a diploid population of size *N*, a neutral mutation has a probability of 1/2*N *of becoming fixed in the population. However, because of drift, even slightly deleterious mutations can become fixed, but at a probability of less than 1/2*N*. Advantageous mutations have higher fixation probabilities than neutral mutations. In the nearly neutral model, the distribution of alleles is determined by an equilibrium of selection, drift, and mutation.

Consider a number of sites under identical evolutionary constraints and with a bias in nucleotide distribution. Assume that weak selection and drift are the causes of this bias; e.g., as for the codon usage biases in micro-organisms and *Drosophila *[9,10]. In our model, some nucleotides confer a slightly higher fitness onto the organism than do others, regardless of their position, and these can become fixed in the population through drift and/or selection. Here, we also assume that selective advantages are additive for the two alleles of the diploid organism [11,12]. Let the selective advantages of the four nucleotides be given by *s*_*A*_, *s*_*C*_, *s*_*G*_, and *s*_*T*_. The differences between these selection coefficients will be very close to zero, since no strong selection is expected.

Consider a mutation from nucleotide *i *to *j*, with a selective advantage of *s *= *s*_*j *_- *s*_*i *_(a selective disadvantage exists when *s *is negative). For a population of size *N *and an effective size of *N*_*e*_, Kimura [11] showed that the fixation probability in this population is given by:



when *s *≠ 0. For *s *= 0, we have *P*(*s*) = 1/2*N*. This approximation is valid for small values of *s*, which is the case here.

The substitution rate from nucleotide *i *to *j *is proportional to *P*(*s*_*j *_- *s*_*i*_):

*q*_*ij *_= 2*N μ*_*ij*_*P*(*s*_*j *_- *s*_*i*_),     (3)

where *μ*_*ij *_is the mutation rate from *i to j*. For different *i *and *j*, *μ*_*ij *_can vary because of unequal transition versus transversion rates (for example). Furthermore, let us assume that the mutation rate is the same for either direction of substitutions between *i *and *j*. This assumption is necessary to maintain the widely used condition of time reversibility in the evolutionary process, which thereby keeps the following derivations tractable [1,13].

**Figure 1:**
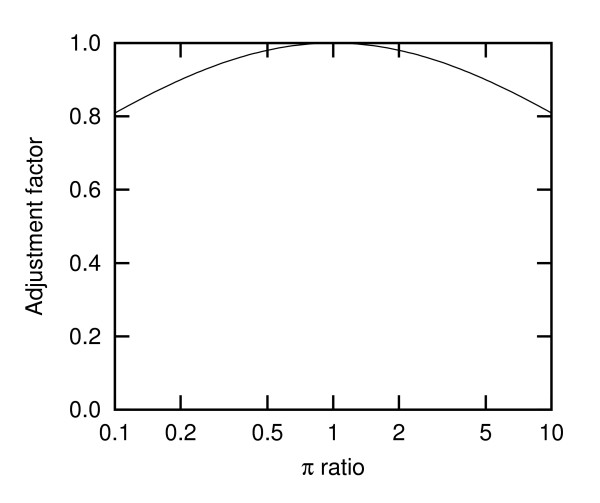
**Adjustment factor as a function of the ratio of *π*'s**. The adjustment factor is given by  (equation (7)).

We then have:



Since *q*_*ij*_/*q*_*ji *_can be written as a function evaluated at *s*_*j *_divided by the same function evaluated at *s*_*i*_, evolution is time reversible according to this model with:



Here, *c *and *c*' are constants with *c*' = -l/4*N*_*e *_log *c*, which will be chosen to make the equilibrium frequencies sum to one. The substitution rates can now be approximated as:



Given an exchangeability of *s*_*ij *_= *μ*_*ij*_, this equation reduces to equation (1) with *f *= 0.5 and an adjustment factor of:



This adjustment factor is close to one for moderate ratios of *π*, with a horizontal tangent around *π*_*j*_/*π*_*i *_= 1 and a slight bending downwards when deviating from this value (Fig. 1). Thus, a value of *f *= 0.5 is suggested for the +gwF variants according to these derivations of the weak selection model.

### Evolutionary simulations

Evolutionary simulations were conducted to examine the effects of violating certain assumptions in the above model of weak selection. Unless otherwise noted, these simulations were based on the K2P+gwF model with *f *= 0.5 and *k *= 2 (for the transition/transversion ratio). Simulations consisted of four sequences of length 10,000 and relied on a symmetric rooted phylogeny with all branch lengths equal to 0.10 expected substitutions per site under the model in question [i.e., ((seq1:0.10, seq2:0.10):0.10, (seq3:0.10, seq4:0.10):0.10)]. Violations of the weak selection model were incorporated in the simulations by: (1) heterogeneous sequences with sites drawn from different equilibrium base frequencies; (2) populations in disequilibrium due to changing *N*_*e*_; and (3) an accelerated C to T substitution rate. Estimates of *f *for the simulated sequences were made with the K2P+gwF model. Forty simulations were run for each test condition, with the results for the *f *estimates summarized as their means and twice their standard errors. In the first set of simulations, six categories of sites with different equilibrium distributions were considered (Table 1). The *f *estimates for the simulations with each category alone were not significantly different from 0.5 (i.e., the value under which the sequences were generated). In contrast, for the simulated heterogeneous sequences (i.e., those composed of equal numbers of sites from two or three different categories), their values of *f *varied significantly in either direction from 0.5. Analogous results were obtained for the simulations of homogeneous and heterogeneous sequences under the HKY model (with *f *= 0.0 instead of 0.5). Thus, the value of *f *can vary considerably when heterogeneous sequences are analyzed with a +gwF model. Here, such deviations are a consequence of using a single rate matrix to analyze sequences that were derived from two or three different ones.

**Table 1:** Starting equilibrium base frequencies and results for the simulations with either homogeneous or heterogeneous sequences (i.e., those with sites from single versus multiple categories, respectively).

Categories		*π*_*C*_	*π*_*G*_	*π*_*T*_	Bias^b^	*f*^c^	
A	0.10	0.40	0.30	0.20	0.154	0.50 ± 0.01	0.00 ± 0.01
B	0.30	0.30	0.30	0.10	0.105	0.50 ± 0.01	0.00 ± 0.01
C	0.30	0.20	0.20	0.30	0.029	0.50 ± 0.02	0.00 ± 0.03
D	0.40	0.20	0.20	0.20	0.078	0.49 ± 0.01	0.01 ± 0.01
E	0.20	0.40	0.20	0.20	0.078	0.51 ± 0.01	-0.01 ± 0.02
F	0.20	0.20	0.40	0.20	0.078	0.50 ± 0.02	-0.01 ± 0.01
A+B	0.20	0.35	0.30	0.15	0.074	0.43 ± 0.01	-0.11 ± 0.02
A+C	0.20	0.30	0.25	0.25	0.015	0.34 ± 0.03	-0.16 ± 0.03
B+C	0.30	0.25	0.25	0.20	0.015	0.24 ± 0.02	-0.33 ± 0.03
A+B+C^e^	0.23	0.30	0.27	0.20	0.016	0.39 ± 0.04	-0.13 ± 0.04
D+E+F^e^	0.27	0.27	0.27	0.20	0.010	0.68 ± 0.03	0.29 ± 0.04

^a^Expected nucleotide distribution.

^b^Nucleotide bias, as information content measured in bits: .

^c^Mean ± twice the standard error of the estimate.

^d^*f *= 0:0 for these simulations with the HKY model. With *f *= 0:0, the HKY+gwF variant is reduced in these simulations to its more standard F81 based model.

^e^The heterogeneous sequences in these simulations were of length 9,999, rather than 10,000, since the latter is not a multiple of 3.

**Figure 2:**
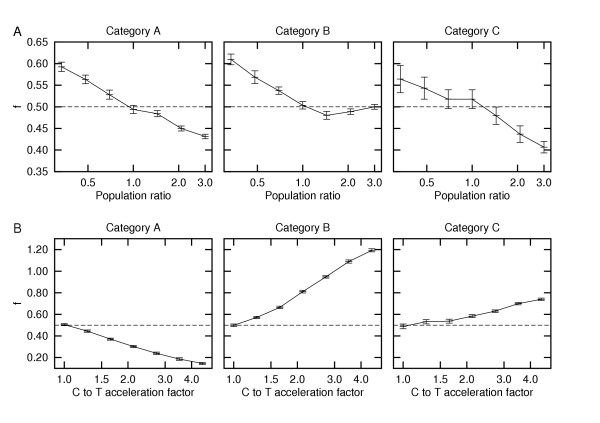
**Two situations where *f *is affected by deviations from the model**. (A) The effect of a change in *N*_*e *_on the value of *f*. This change in *N*_*e *_occurs in the most recent common ancestor of the four simulated sequences. Population ratio refers to its *N*_*e *_after versus before this change. (B) The effect of an increased C to T substitution rate. Categories A, B, and C are defined in Table 1.

In the second set of simulations, *N*_*e *_was kept constant until the time of the most recent common ancestor for the four simulated sequences. Then, *N*_*e *_was either left unchanged or was suddenly changed by a certain factor. The latter was done by replacing the rate matrix derived from equation (4), resulting in new equilibrium frequencies of the nucleotides. When *N*_*e*_was kept constant, the selective pressures and drift were left unchanged, thereby maintaining the same starting equilibrium frequencies throughout the phylogeny. Thus, the corresponding *f *estimates did not significantly differ from 0.5 (Fig. 2). In contrast, increases in *N*_*e *_lowered the value of *f *as the efficiency of selection was increased relative to drift [4]. Correspondingly, the evolutionary process became more dominated by the ending nucleotide. This increasing dominance can be expected to continue until a new equilibrium is restored (which occurs on a longer time scale than that in these simulations).

In the third set of simulations, an acceleration in the C to T substitution rate was incorporated, thereby modeling an increase in their mutation rate due to the deamination of methylated C's in CpG pairs [14]. The introduction of this bias resulted in significant deviations of *f *in either direction from 0.5, even though their sequences were simulated in equilibrium (Fig. 2). Thus, the value of *f *can vary considerably when the rates for reciprocal mutations are unequal.

## Discussion

This study illustrates how selection, drift, and mutation within a population can be linked to the *f *parameter and rate matrices of the +gwF variants for sequence evolution. Our weak selection model relies on the fixation probabilities of mutant alleles with additive genie selection and equal mutation rates for reciprocal substitutions. What is now needed are additional studies that link other population genetics models to the +gwF variants [9]. For example, the population genetics models of Li [15], which focus on allele frequency distributions and different modes of selection and mutation, could be studied for their connections to the *f *parameter and +gwF rate matrices.

Collectively, the three sets of simulations highlight that the *f *parameter is complex and can be influenced by a number of different factors [4]. This complexity limits its biological interpretation and the use of its expected value of 0.5 as derived for the weak selection model. Correspondingly, in many +gwF analyses, *f *ill need to be estimated as a free parameter rather than fixed beforehand.

Goldman and Whelan [4] focused on amino acid sequences, where they found that the +gwF models provided better fits to the majority of their protein data sets. They also analyzed two rather small nucleotide data sets for which the general reversible model (REV) outperformed the +gwF variants. As noted by them, the REV model provides enough free parameters to cover the effects of a +gwF analysis. Thus, given sufficient data, this model will consistently outperform the simpler +gwF variants, since it can always accommodate more of the evolutionary process by virtue of its extra parameters. Nevertheless, as widely acknowledged, simpler models have their place, since they allow one to maximize analytical power for more limited data, while minimizing the risk of over-parameterization [13,16]. Thus, as for the JC, K2P, and HKY models, we expect their +gwF variants to remain of interest as part of the hierarchy of simple to complex models for sequence evolution.

## Authors' contributions

Both authors contributed to the conception and design of this study and to the writing, reviewing, and final approval of this article. B.K. performed the simulations and parameter estimations.
